# A Comprehensive Analysis of Diversity, Structure, Biosynthesis and Extraction of Biologically Active Tannins from Various Plant-Based Materials Using Deep Eutectic Solvents

**DOI:** 10.3390/molecules29112615

**Published:** 2024-06-02

**Authors:** Maja Molnar, Martina Jakovljević Kovač, Valentina Pavić

**Affiliations:** 1 Faculty of Food Technology Osijek, Josip Juraj Strossmayer University of Osijek, F. Kuhača 18, 31000 Osijek, Croatia; mmolnar@ptfos.hr (M.M.); mjakovljevic@ptfos.hr (M.J.K.); 2 Department of Biology, Josip Juraj Strossmayer University of Osijek, Cara Hadrijana 8/A, 31000 Osijek, Croatia

**Keywords:** deep eutectic solvents, extraction, tannins, proanthocyanidins, hydrolysable tannins, gallotannins, ellagitannins

## Abstract

This paper explores the emerging subject of extracting tannins from various plant sources using deep eutectic solvents (DESs). Tannins are widely used in the food and feed industries as they have outstanding antioxidant qualities and greatly enhance the flavor and nutritional content of a wide range of food products. Organic solvents are frequently used in traditional extraction techniques, which raises questions about their safety for human health and the environment. DESs present a prospective substitute because of their low toxicity, adaptability, and environmental friendliness. The fundamental ideas supporting the application of DESs in the extraction of tannins from a range of plant-based materials frequently used in daily life are all well covered in this paper. Furthermore, this paper covers the impact of extraction parameters on the yield of extracted tannins, as well as possible obstacles and directions for future research in this emerging subject. This includes challenges such as high viscosity, intricated recovery of compounds, thermal degradation, and the occurrence of esterification. An extensive summary of the diversity, structure, biosynthesis, distribution, and roles of tannins in plants is given in this paper. Additionally, this paper thoroughly examines various bioactivities of tannins and their metabolites.

## 1. Introduction

Tannins, a diverse group of polyphenolics, play crucial roles in the plant kingdom and have extensive applications in the food and feed industries [1,2], due to their potent anti-inflammatory, antibacterial, and antioxidant properties. These applications enhance food quality, prolong shelf life, and contribute to animal wellbeing [3,4]. However, the complex chemical structures and diverse distribution of tannins within plants pose significant challenges for efficient extraction, a critical step in utilizing these valuable natural resources [5].

Recent advancements in green chemistry have highlighted deep eutectic solvents (DESs) as an attainable alternative to traditional organic solvents for extracting bioactive compounds, including tannins [6,7]. DESs offer numerous advantages, such as minimal toxicity, biodegradability, low volatility, enhanced solvation properties, and adjustable characteristics, which make them particularly suited for environmentally sustainable and efficient extraction processes. Their exceptional capacity to dissolve an extensive array of substances, such as tannins, in moderate circumstances renders them especially well-suited for environmentally friendly extraction methods [8]. This thorough analysis aims to investigate the possibilities of using deep eutectic solvents in the extraction of tannins from plant-based products in food and feed applications.

We will discuss the chemical properties of tannins and their distribution in plants. In addition, we will give a general review of deep eutectic solvents, covering their composition, characteristics, and uses in tannin extraction. We will also discuss current developments in the application of DESs for tannin extraction and provide valuable insights into the use of deep eutectic solvents as effective and sustainable tannin extraction media, providing a pathway for the development of novel approaches in food and feed applications by synthesizing current research findings and identifying key challenges and opportunities.

In addition to their use in food and feed, tannins exhibit an array of bioactivities. The medicinal attributes of tannins, such as their antibacterial, anti-inflammatory, antioxidant, and anticancer effects, are becoming more widely acknowledged [9,10,11]. The complex chemical structures of tannins, which enable interactions with a variety of biological targets and pathways, are the source of these bioactivities. The ability of DESs to effectively extract tannins not only supports industrial applications, but also opens new avenues for research into the medicinal uses of tannins. By synthesizing current findings and identifying ongoing challenges and opportunities, this review aims to provide a comprehensive overview of the potential of deep eutectic solvents in enhancing the accessibility and usability of tannins from natural sources. In summary, this review highlights the significance of analyzing the various bioactivities of tannins and their metabolites, indicating their potential as versatile substances with benefits to human health and welfare.

## 2. Tannins: Diversity, Structure and Distribution in Plants

**Figure 1:**
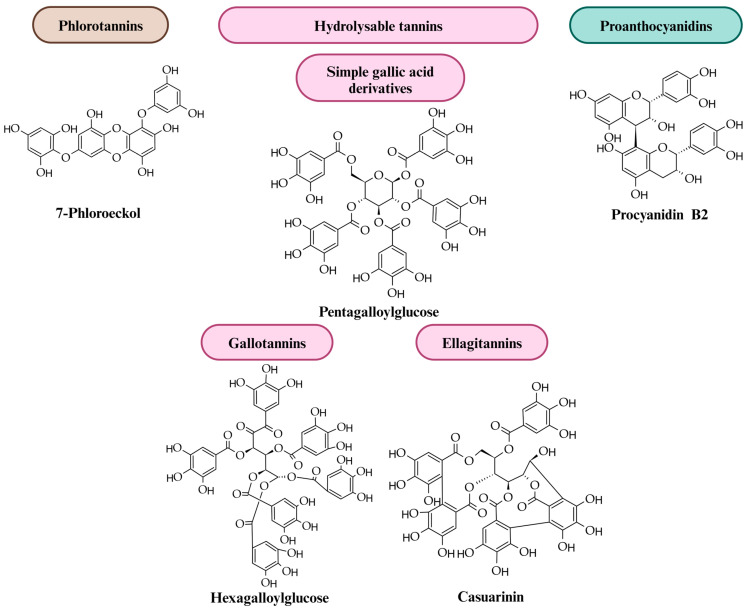
Some examples of structures of the tannin main subgroups.

Tannins, a diverse group of naturally occurring polyphenolic compounds found in various parts of plants, can be broadly categorized into two main groups: hydrolysable tannins (HTs) and proanthocyanidins, also called condensed tannins (CTs). Phlorotannins (PTs) are being reported in the literature as a third major class of tannins [12]. Every group has unique biological roles and structural characteristics (Figure 1). Phlorotannins are the most basic tannin group structurally, and they are primarily found in aquatic species, such as brown algae [13]. Phloroglucinol (an aromatic ring with 1,3,5 hydroxyl groups) units (≥2) are the building blocks of single phlorotannins, which are linked by C–O–C or C–C bonds to form oligomers, such as tetrameric phlorotannin. Additional hydroxyl groups in the molecules or bonds between the monomers and the higher presence of phloroglucinol subunits (3 to 7 subunits) leads to structural variations [5]. They are grouped into three distinctive classes based on the coupling between subunits: fucols (C–C), phloroetols (C–O–C), and fucophloroteols (C–C and C–O–C). This description of phlorotannins based on structure makes a very obvious distinction between them and other phenolic compounds.

The molecular weights of hydrolysable tannins range from the simple glucogallin (MW 332) to pentameric ellagitannins, with MWs over 5000 Daltons (Da) [14]. Proanthocyanidins are oligomeric or polymeric flavan-3-ols with molecular weights from 500 (two monomeric units) to over 23,900 Da, with 83 monomer units as shown by Cheynier et al. [15]. The broad spectrum of hydrolysable tannin compositions, resulting from the combination of glucose and gallic acid, is the cause of the various divisions and subdivisions found in the literature [9,16,17,18,19]. Upon hydrolysis, hydrolysable tannins yield gallic acid or ellagic acid and sugars as degradation products [20]. Two subclasses of hydrolysable tannins are distinguished by their structural differences: ellagitannins (ETs) and gallotannins (GTs), which are formed from the esters of ellagic acid or gallic acid, respectively, and are linked to a sugar moiety, such as glucose [17]. Many researchers consider that this division of the tannins, although the most often accepted, does not accurately reflect their chemical complexity. Thus, depending on their structural characteristics, hydrolysable tannins can be categorized into at least three subclasses: simple gallic acid derivatives, gallotannins, and ellagitannins [12,18]. Simple gallic acid derivatives contain five or less galloyl groups which are most frequently esterified to quinic acid or glucose (from monogalloyl and pentagalloyl glucose) [21]. As the structures do not show an abundance of variations, the most frequently occurring compounds are tetra- and penta-*O*-galloyl-glucoses, so 1,2,3,4,6-penta-*O*-galloyl-β-d-glucose (PGG) is the group’s most researched compound [17]. Gallotannins are natural polymers that possess a core structure in which a carbohydrate, mainly glucose, or cyclohexanecarboxylic acid (quinic or shikimic) is galloylated at hydroxyl groups. They are further distinguished by the presence of six or more digalloyl groups (heptagalloyl glucose). GTs are characterized by the presence of depsidically linked galloyl units [16] formed by the esterification of D-glucose hydroxyl groups and gallic acid in polymeric chains, where the galloyl moieties are linked via “depside” bonds (ester bond in polyphenols composed of two or more monoaromatic units) [12,22]. 

Ellagitannins (ETs) are derived from the esters of hexahydroxydiphenic acid, which forms ellagic acid as a lactone, and polyols, such as glucose or quinic acid. Ellagitannins are formed through the oxidative coupling of galloyl groups to produce hexahydroxydiphenoyl (HHDP) groups, which are biarylic dehydrodigalloyls. This is what distinguishes the ellagitannins. According to one of the most basic descriptions of ETs, when the HHDP group is hydrolyzed, the ET molecule spontaneously rearranges to form insoluble ellagic acid. In plants, methylation, glycosylation, and methoxylation result in a variety of ET derivatives [23]. C-glycosidic ETs can be described as castalagin-type and casuarinin-type. A flavogalloyl moiety is connected to the *C*-glycosidic fraction in castalagin-type compounds, which results in compounds like vescalagin and castalagin. However, in casuarinin-type ETs, the C-glycosidic linkage includes an HHDP unit, resulting in casuarinin and stachyurin [24]. The unique structure of C-glycosidic ellagitannins includes a C–C linkage between carbon-1 of an open-chain glucose core and carbon-2′ of a galloyl-derived unit. This unit can be part of a terarylic nonahydroxyterphenoyl (NHTP) group attached via ester bonds to the 2-, 3-, and 5-positions of the glucose core, as in vescalagin and castalagin, or a biarylic variant bridging the 2- and 3-positions of the glucose core, as in stachyurin and casuarinin [25]. However, this is too simplified, as ETs are different from the other tannin classes based on their structures [26]. Six subgroups can be identified among the various ET structures that have been described so far (more than 500): hexahydroxydiphenoyl (HHDP) esters, dehydro-HHDP esters and their modifications, nonahydroxytriphenoyl (NHTP) esters, flavonoellagitannins, and oligomers with different types of bonds between the monomers, as well as varying degrees of oligomerization [26]. 

**Figure 2:**
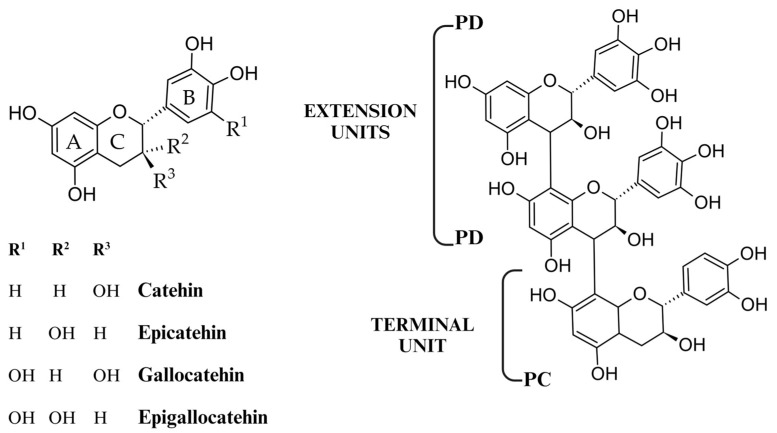
Example of a trimeric proanthocyanidin. The extension units are gallocatechin and epigallocatechin (PD), and terminal unit catechin (PC). Adapted from Engström 2016 [29].

Proanthocyanidins are polyhydroxy non-branched [3,27] oligomers and polymers of flavan-3-ols, mostly (−)-epicatechin (extension unit) and (+)-catechin units (terminal unit) [28,29], joined by C–C linkages that are not susceptible to hydrolysis, as shown in Figure 2. [30,31,32]. Given that proanthocyanidins degrade down oxidatively, resulting in reddish anthocyanin pigments when heated in acidic conditions, they are known as proanthocyanidins (PAs) [33]. The enzyme anthocyanidin reductase converts anthocyanidins into epicatechins [34,35]. Because proanthocyanidins are composed of phenols of the flavone type, polymers of flavan-3,4-diols like leucocyanidins, or flavan-3-ols like catechin, they are also often referred to as flavolans. Unlike the hydrolysable tannins, they lack in sugar residues [36]. The C4/C8 bond is the primary connection that connects the flavan-3-ol units; however, there is also a C4/C6 bond (both are referred to as B type). Another possible way to double-link the flavan-3-ol units is to add an extra ether bond between C2/O7 (A type). The hydroxylation pattern, interflavan linkages, and degree of polymerization of the proanthocyanidins can be used to characterize them [37,38]. Based on the latter, three frequent flavan-3-ols have been identified: procyanidins (PC), which are solely composed of (epi)catechin subunits; propelargonidins, which are composed of (epi)afzelechin subunits; and prodelphinidins (PD), which are composed of (epi)gallocatechin subunits [26,39,40]. Barbehenn et al. [41] demonstrated that PD-rich polymers possess the ability to prevent ET oxidation at high pH in vitro, while PC oligomers and polymers were less successful. The real structures of all individual CT oligomers and polymers in a plant sample are quite difficult to determine, as might be expected even from the numerous monomeric units. Nonetheless, it is possible to easily ascertain the mean degree of polymerization and the average nature of the monomeric building blocks (PC:PD ratio) [42,43].

**Figure 3:**
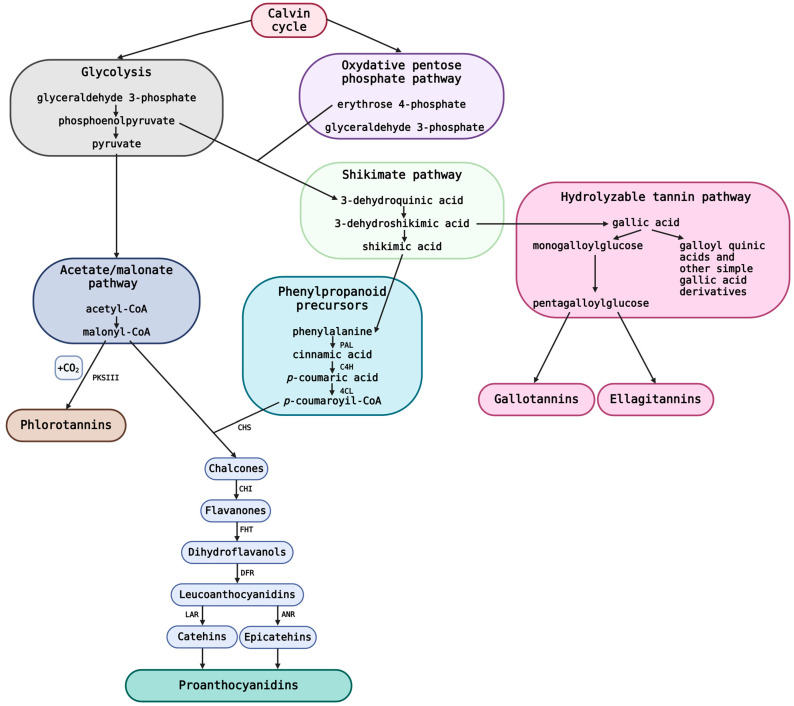
The biosynthetic pathways leading to phlorotannins, hydrolysable tannins, and proanthocyanidins (adapted from [26,29], created with BioRender.com (accessed on 26 May 2024) [48]). PKSIII: type III polyketide synthase, PAL: phenylalanine ammonia-lyase, C4H: cinnamic acid 4-hydroxylase, 4CL: 4-coumarate-CoA ligase, CHI: chalcone isomerase, CHS: chalcone synthase, FHT: flavanone 3β-hydroxylase, DFR: dihydroflavonol 4-reductase, LAR: leucoanthocyanidin reductase, and ANR: anthocyanidin reductase.

Cell walls have been proposed as a typical site for the biosynthesis and accumulation of hydrolysable tannins [44]. Three different pathways lead to the production of tannins: the phenylpropanoid pathway, the flavonoid pathway, and the shikimic acid pathway (for shikimic acid) [45]. The biosynthesis pathways that result in HTs and PAs are schematically shown in Figure 3. Carbohydrates from the Calvin cycle, such as sucrose and starch, are converted in the initial stages of polyphenol biosynthesis into either glyceraldehyde-3-phosphate via glycolysis or erythrose-4-phosphate and glyceraldehyde-3-phosphate via the oxidative or reductive pentose phosphate pathway [26]. The acetate/malonate pathway and the shikimate pathway are the first sites where HT and PA biosynthesis differ; whereas PA synthesis makes use of both pathways, HT production only uses the shikimate pathway [26]. The synthesis of pyruvate required for the acetate/malonate route is greatly decreased if substantial amounts of glycolytic phosphoenolpyruvate are directed towards the shikimate pathway (along with erythrose-4-phosphate). As one of their building ingredients would be malonyl-CoA, this directly hinders the formation of PAs. Phlorotannins are synthesized through a malonate/acetate pathway, catalyzed by type III polyketide synthase (PKSIII). This process involves the condensation of malonyl-CoA units with carbon dioxide, leading to phloroglucinol isomers. Further chemical changes, like the Claisen cyclization of malonyl-CoA, occur, produces thermally stable forms [46]. Tautomerization of these compounds yields various phlorotannins with different carbon–carbon and carbon–oxygen linkages [47]. However, while the phlorotannin-producing brown algae only require the acetate/malonate route for their tannin synthesis, all plant tannins depend on the effective operation of the shikimate system in particular [26]. 

The spot of the second key branch point is 3-dehydroshikimic acid. This is the starting point for the production of gallic acid, which is the main component of all HTs. The synthesis of shikimic acid and its byproducts, PAs, flavonoids, and derivatives of caffeic and coumaric acids, is adversely affected by the efficient production of gallic acid. A third important branch point in the HTs route can be found at pentagalloyl glucose, which is the precursor to both GTs and ETs. Significant levels of both of these HT classes have not yet been reported to be produced simultaneously in the same plant tissue. Furthermore, it has only been reported that coniferous plants synthesize PAs, but never HTs [49]. As pentagalloylglucose galloylates into hexa-, hepta-, octagalloylglucose, etc., the biosynthesis pathway yields GTs [50,51]. Unlike GT biosynthesis, the putative ET pathway is largely speculative, because only two of its stages have been thoroughly investigated by enzymatic research [51,52,53,54]. 

One of the numerous branches of the biosynthetic pathways produces PAs. The amino acid phenylalanine, which is synthesized by the shikimate pathway and changes into *p*-coumaroyl-CoA via the phenylpropanoid pathway, is thought to be the source of both the aromatic B-ring and the three carbon atoms of the heterocyclic C-ring [55,56]. The shikimate pathway is the starting point for the production of phenylpropanoids and a source of phenylalanine. Three enzymatic activities characterize the so-called central phenylpropanoid pathway: (i) phenylalanine deamination to *trans*-cinnamic acid by phenylalanine ammonia-lyase (PAL); (ii) *trans*-cinnamic acid hydroxylation to *p*-coumaric acid, resulting from the activity of cinnamic acid 4-hydroxylase (C4H); and (iii) 4-coumarate conversion to the 4-coumaroyl-CoA by 4-coumarate-CoA ligase (4CL) [57]. These intermediates serve as precursors for the synthesis of various phenolic compounds, including tannins.

Three units of malonyl-coenzyme A (malonyl-CoA), which are created by the acetate/malonate pathway, combine to form the A-ring [55,58]. When the C-ring closes, it turns into flavanone naringenin, which then converts into dihydrokaempferol when a hydroxyl group is added to the C3 position of the C-ring by flavanone 3β-hydroxylase (FHT) [59]. By reducing these dihydroflavonols by dihydroflavonol 4-reductase (DFR), leucoanthocyanidins (flavan-3,4-diols) and flavan-3-ols, such as catechin and gallocatechin, are produced [30,60]. The other possibility is the oxidation of the leucoanthocyanidin molecules to produce anthocyanidins [45,59], which can then be transformed into the flavan-3-ols epicatechin and epigallocatechin [45,61]. It is possible for flavan-3-ols (catechins) and flavan-3,4-diols (leucoanthocyanidins) to serve as precursors for PAs [35,61,62] through the action of enzymes such as leucoanthocyanidin reductase (LAR) and anthocyanidin reductase (ANR). LAR converts leucoanthocyanidin into catechin, while ANR converts anthocyanidin into epicatechin [34,63,64]. Alternatively, flavan-3-ols can be polymerized into proanthocyanidins by the action of enzymes such as anthocyanidin reductase (ANR) and/or leucoanthocyanidin dioxygenase (LDOX). However, the mystery surrounding the polymerization phase of PAs has persisted [35,45,65]. 

Due to the shared building blocks in the biosynthesis of PA and HT, their formation could be in competition. The production of pyruvate, which is used in the acetate/malonate pathway, is greatly decreased if the glycolytic phosphoenolpyruvate is effectively directed into the shikimate pathway. As a result, there is less PA biosynthesis, since malonyl-CoA is required for one of its building blocks [26]. Comparably, efficient gallic acid synthesis from 3-dehydroshikimic acid reduces PA biosynthesis, as it negatively impacts the synthesis of shikimic acid, which in turn prevents the development of phenylalanine, the second PA precursor [26]. A similar situation holds for the two HT classes, GTs and ETs; this is likely because of their shared precursor, pentagalloylglucose. 

The biosynthesis and regulation of tannins in plants involves complex molecular mechanisms that are tightly controlled by genetic, environmental, and developmental factors. Understanding tannin production can help explain why various species’ tissues and tannin contents differ, as well as their eventual effects on ecology and evolution. While HTs tend to be localized in the cell walls of plant tissues [21,44], PAs appear to accumulate in the vacuole [35,60,65]. Also, a normal plant cell will not produce ETs and GTs at the same time, as the effective synthesis of ETs negatively impacts proanthocyanidins. Because of this, ETs and PAs do not both accumulate in high concentrations in the same tissue. Instead, their contents usually exhibit opposite seasonal trends, with ETs peaking in young tissues, and PAs being most prevalent in mature leaves [49,66,67]. 

The biosynthesis of tannins is regulated at multiple levels, including via transcriptional, post-transcriptional, and post-translational regulation. The role of transcriptional regulation in the phenylpropanoid pathway has been the leading model for studies on plant gene regulation [68,69,70,71,72]. Transcription factors such as MYB, bHLH, and WD40 proteins regulate the expression of genes involved in tannin biosynthesis by binding to specific cis-acting elements in the promoter regions of target genes [63,64,65,66,67,68,69]. Environmental factors, such as light, temperature, water availability, and nutrient status, also influence tannin biosynthesis by modulating the expression of key regulatory genes and enzymes [45,73]. Blood oranges under cold stress can express more flavonoid genes and have higher quantities of flavonoids as a result [74]. Visible light yields proanthocyanidins, whereas ultraviolet light stimulates anthocyanin synthesis after immature grape fruits are exposed to both types of light [75]. Overall, the biosynthesis and regulation of tannins in plants involves intricate molecular mechanisms that coordinate the expression of biosynthetic genes and the activity of enzymatic pathways in response to endogenous and environmental cues. 

Genetics, habitat, and developmental phases are only a few of the influencing factors that can cause significant variations in tannin concentration both within and between plant species. Tannins are commonly found in numerous plant tissues, including the roots, wood, bark, leaves, and fruits, such as bananas, blackberries, apples, and grapes [76,77,78,79]. They are especially abundant in the bark of conifers, oaks, sumacs, and myrobalan [12,80]. About half of the 99 food plant species examined in the study carried out by Miljković et al. [1] had tannins, most of which were berries and fruit; tannins were not evident in vegetables. However, some vegetables may contain low levels of tannins, particularly those belonging to plant families known to produce them, such as the Fabaceae family. They can also be found in galls, which are abnormal growths caused by insect attacks [81,82]. Within the plant kingdom, tannins are found in a wide range of plant families and species. As they provide defense against pathogens and herbivores, they are especially prevalent in woody plants. Tannin-free species can be found in the majority of dicot families (tested by their ability to precipitate proteins). Aceraceae, Actinidiaceae, Anacardiaceae, Bixaceae, Burseraceae, Combretaceae, Dipterocarpaceae, Ericaceae, Grossulariaceae, Myricaceae, Najadaceae, and Typhaceae are the best-known families in which all species tested contain tannins. In the Fagaceae family, 73 percent of the oak species tested contained tannins [83]. 

Phlorotannins are primarily found in aquatic species, such as brown algae (Table 1). GTs are commonly found in certain berries, grapes, persimmons, and pomegranate [17]. Compared to ETs, simple gallic acid derivatives and GTs are far less common in plants [26]. Cashew nuts, pistachios, mangos, hazelnuts, persimmons, chestnuts, walnuts, guavas, cloves, pimento, pomegranates, plums, apricots, peaches, bird cherries, strawberries, raspberries, blackberries, blackcurrants, gooseberries, teas, grapes, and muscadine grapes are among the plant species that contain ETs [17]. Vescalagin is an example of an ellagitannin derived from *β*-pentagalloylglucose [84], found in pomegranate peels [85], oak [86], and chestnut wood [87], and in the stem barks of African birch [88]. PAs are widely distributed and, after lignin, are the second most prevalent natural phenolic [37]. Proanthocyanidins have been identified in numerous dicotyledonous and monocotyledonous flowering plants, gymnosperms, and fern allies [39,89]. In contrast, HTs are only found in dicotyledonous plants [9,90]. Many plants’ fruits, bark, leaves, and seeds contain proanthocyanidins, as do many foodstuffs made from plants, including green tea, apples, cocoa, chocolate, grapes, apricots, and cherries [2]. Red wine and fruit juices are particularly high in proanthocyanidins [91]. Red wine and tea have an astringent flavor because of the presence of proanthocyanidins in them. Plants like grape (*Vitis vinifera* L.), cacao tree (*Theobroma cacao* L.), and cranberry (*Vaccinium macrocarpon* L.) have significant quantities of them [40,92,93]. Other plant sources of different tannins are presented in Table 1.

**Table 1:** Some plant sources of different types of tannins.

Plant Material	Compounds	Reference
Brown alga (*Ecklonia kurome* Okamura)	Eckol	[94]
Paddle weed (marine brown alga) (*Ecklonia cava* Kjellman)	Fucodiphlorethol G Phlorofucofuroeckol A Phloroglucinol-6,6′-bieckol	[95,96,97]
Walnut seeds (*Juglans regia* L.)	Pedunculagin Casuarictin Tellimagrandin I, II Glansreginin A, B Casuarinin	[98,99,100]
Mango peel (*Magnifera indica* L.)	Methyl gallate Maclurin 3-C-β-d-glucoside Iriflophenone 3-C-β-d-glucoside Tetra-*O*-galloyl-glucoside Penta-*O*-galloyl-glucoside Mangiferin Maclurin 3-*C*-(2-*O*-galloyl)-β-d-glucoside	[101]
Mango bark (*Magnifera indica* L.)	Gallic acid Methyl gallate Maclurin 3-C-β-d-glucoside Iriflophenone 3-C-β-d-glucoside Isomangiferin Iriflophenone 3-C-(2-*O*-galloyl)-β-d-glucoside Penta-O-galloyl-glucoside Mangiferin	[101]
Mango old leaves (*Magnifera indica* L.)	Gallic acid Methyl gallate Maclurin 3-C-β-d-glucoside Iriflophenone 3-C-β-d-glucoside Penta-*O*-galloyl-glucoside Iriflophenone 3-C-(2-*O*-Galloyl)-β-d-glucoside	[101]
Mango young leaves (*Magnifera indica* L.)	Gallic acid Methyl gallate Maclurin 3-C-β-d-glucoside Iriflophenone 3-C-β-d-glucoside Tetra-*O*-galloyl-glucoside Penta-*O*-galloyl-glucoside Iriflophenone 3-C-(2-*O*-galloyl)-β-d-glucoside	[101]
Evening Primrose *(Oenothera erythrosepala* Borbás)	Oenothein B	[102]
Garden Spurge (*Euphorbia hirta* L.)	Euphorbin A	[103]
Japanese cornelian cherry (*Cornus officinalis* Torr.)	Cornusiin A	[102]
Common Reaumuria (*Reaumuria hirtella* Jaub. and Spach.)	Hirtellin A	[104]
Hairy Agrimony (*Agrimonia pilosa* Ledeb.)	Agrimoniin	[105]
Autumn Olive (*Elaeagnus umbellata* Thunb.)	Casuglaunin A	[106]
Thiloa leaves (*Thiloa glaucocarpa* Eicher.)	Vescalagin	[107]
Blackberry fruits (*Rubus fruticosus* L.)	Sanguiin H-6 Lambertianin C	[108,109]
Raspberry fruits (*Rubus idaeus* L.)	Sanguiin H-6 Lambertianin C	[110]
Pomegranate fruits and peels (*Punica granatum* L.)	Punicalagin Punicalin Pedunculagin Vescalagin Castalagin Casuarin Granatin B Oenothein B Eucalbanin B Eucarpanin T1 Pomegraniins A, B	[111,112,113,114,115]
Jaboticaba seeds (*Plinia cauliflora* (Mart.) Kausel)	Pedunculagin	[116]

Tannins are more prevalent in plant tissues that are vulnerable, like young leaves and blossoms [117]. The biological activities of various extraction sources vary, causing plant species, growth phases, and growth environments to all have significant influence in plant tannin chemical structures and contents [118,119]. However, genetics, habitat, and developmental phases are only a few of the influencing factors that can cause significant variations in tannin concentration, both within and between plant species. Different fruit varieties and cultivars may have significantly different tannin content [120]. Some fruits, such as persimmons, have both high- and low-CT variants. Tannin-rich fruits accumulate tannins until they are fully grown, which lends a disagreeable flavor, while low-tannin fruits stop producing PAs at a fundamental stage of growth [121]. Changes in seed phenolics were linked to berry development and maturation in *Vitis vinifera* L. cv Shiraz [122]. 

Tannins have a unique ability to form complexes with proteins due to hydrophobic interactions and hydrogen bonding. This precipitation of proteins occurs when phenolic groups in tannins bond with carboxyl groups in proteins. Consequently, tannins tend to accumulate in vacuoles or on cell surfaces to avoid interference with plant metabolism [28,123,124,125]. These vacuoles play a crucial role in controlling tannin activity, as they prevent tannins from accessing functional proteins in the cytoplasm until cell breakdown occurs. Tannins are believed to be synthesized outside vacuoles and then transported into the vacuole lumen [31,65,126]. Research on honeysuckle petal trichomes conducted by Qu et al. [28] revealed a complex network of multiply folded membranes surrounding tannin-accumulating vacuoles. These membranes extend from the vacuole periphery into the cytoplasm, suggesting a close association between tannin accumulation and membrane structures. This structural arrangement likely enhances the efficiency of vacuolar tannin accumulation.

The movement of proanthocyanidins production from the abaxial (lower) to the adaxial (upper) side of the leaf during growth is part of a process known as differentiation and specialization [84]. Since it concentrates these molecules where they could be most needed for protection against herbivory or other environmental challenges, it can be considered a part of the plant’s defense mechanism. Several metabolic reactions occur during leaf development to create secondary metabolites such as proanthocyanidins. During leaf development, specialized cells called idioblasts form in the epidermis, and play a crucial role in storing and sometimes secreting these compounds. The results of the bibliographic review carried out by Ribeiro et al. [127] showed that idioblasts, hypodermis, and epidermis are just a few of the many types of cells in the Rhamnaceae family that can retain mucus. Tannin-secreting cells can be found in the mesophyll, epidermis, and particularly in the parenchymal sheath surrounding vascular bundles in the majority of species. Some taxa in this family, like *Helinus* (Gouanieae), *Noltea*, *Phylica* (Phyliceae), *Krugiodendron*, and *Rhamnus* (Rhamneae), solely contain tannin idioblasts instead of mucilage. 

Tannins contribute to the structural defense of plant tissues by reinforcing cell walls and forming physical barriers against pathogen penetration. They can also promote the deposition of lignin and suberin, further strengthening plant defenses. They seem to be accumulated in the walls of cortical cells after cell death [128], which may serve as a barrier against pathogens and decay organisms. This process is particularly important in woody plants, where lignification and tannin accumulation contribute to the formation of durable and resistant tissues, such as bark and wood. Tannins play essential roles in plant defense mechanisms against herbivores, pathogens, and environmental stresses [129,130,131]. Tannins possess antimicrobial properties, inhibiting the growth and proliferation of bacteria, fungi, and other pathogens. They achieve this by disrupting microbial cell membranes, interfering with enzyme activity, and chelating metal ions essential for microbial growth [132]. While defense against herbivory and pathogens remains a primary function of tannins, stress-induced tannin accumulation may suggest that tannins have other purposes in vegetative tissue, including leaves. This is particularly true for the quaking aspen (*Populus tremuloides* Michx.), where damage and insect herbivory cause leaf CT levels to increase [133]. Rubert-Nason et al. [133] reported how foliar CT concentrations, polymer sizes, representation of procyanidins and prodelphinidins, and stereochemistry vary in response to changes in air temperature (warming and freeze damage), air composition (elevated CO_2_ and O_3_), soil quality (nutrients and microbiome), and herbivory (mammal and lepidopteran). All environmental variables explored, except for soil microbiome, have been found to influence both CT quantity and quality, with climate factors appearing to have larger effect magnitudes than herbivory. Climate, soil, and herbivory effects varied among genotypes, while air composition effects were consistent across genotypes. Tannins possess antioxidant properties, meaning they can help scavenge reactive oxygen species (ROS) produced during stress conditions such as drought, high temperatures, or nutrient deficiencies. By reducing oxidative damage, tannins contribute to the plant’s ability to withstand stress [134]. Tannins can absorb UV radiation, providing protection to plant tissues from UV-induced damage. Under conditions of increased UV exposure, such as high-altitude environments or periods of intense sunlight, tannins may accumulate to provide additional protection to the plant [135]. They also provide protection against drought [136], extreme temperatures [137], and have a high affinity for binding metal ions [138]. Tannins released from plant tissues into the soil can inhibit the germination and growth of pathogenic microbes and competing plant species, exerting allelopathic effects [139]. 

Tannins also have regulatory roles in various aspects of plant growth and development. These regulatory functions are mediated by tannin interactions with hormonal pathways, nutrient dynamics, and physiological processes within plants [12]. Tannins can influence seed germination by acting as germination promoters (low concentration) or inhibitors (high concentrations), depending on their concentration and chemical properties [140,141]. They accumulate in developing seeds, where they contribute to seed coat formation, embryo protection, and seed dormancy regulation. Also, an influence on seed quality, viability, and germination potential has been observed [142]. In some cases, tannins released into the soil may inhibit the germination and growth of sensitive plant species, while promoting the growth of tolerant species [143,144]. In some cases, tannins promote lateral root development and root hair formation, facilitating nutrient and water uptake from the soil [145]. However, high concentrations of tannins may inhibit root growth and elongation, leading to reduced root biomass and impaired nutrient acquisition [146]. Tannins may also influence flowering time and floral development through their effects on hormone metabolism, photoperiodic responses, and nutrient allocation [147]. They often interact synergistically with other antioxidants, such as flavonoids, phenolic acids, and carotenoids, to enhance the plant’s antioxidant capacity and defense against oxidative stress [148]. Overall, tannins play significant roles in the context of climate change, affecting plant responses to changing conditions and ecosystem dynamics. Tannin-rich plants may exhibit greater tolerance to environmental stressors and competitive advantages under changing climatic conditions [149].

## 3. Deep Eutectic Solvents

Deep eutectic solvents have drawn the attention of many researchers since they were investigated by Abbot at al. in 2003 [150]. Their formation, characterized by an intense decrease in melting point as compared to the pure components, was attributed to the formation of net of hydrogen bonding, van der Waals forces, and other intermolecular interactions formed within the solvents [151,152,153,154,155]. In fact, all of their physical and chemical properties are governed by the type and ratio of components and their intermolecular interactions [154,156]. These days, DESs are designated in five groups [157], depending on the components. They can also be classified as NADES, which are formed of purely natural compounds, THEDES, formed of pharmaceutically active compounds, or TDES, ternary DESs formed of three components. Since their physical and chemical properties vary greatly depending on the combined components, they can be designed or tailored for specific uses. This is also one of their great advantages, followed by biodegradability, low vapor pressure, environmental friendliness, and in most cases, low or non-toxicity [158,159]. However, it is important to state that all of these properties vary greatly depending on their composition [160]. They are usually described as non-toxic, but some research has revealed that the non-toxicity of pure components does not indicate the non-toxicity of DESs and vice versa, i.e., some pure components show higher individual toxicity than when they are combined into a DES [159,161,162,163]. One of their main disadvantages, which limits their broad application in industry, is their high viscosity [158], which can be decreased to some extent by the addition of water. However, the addition of water can influence the structure of DESs, and if added above the limit, which is characteristic to each DES, it can disrupt the structure, turning the whole mixture into an aqueous solution [164]. Nevertheless, DESs are easy to handle and prepare, which includes the mixing of components at an exact ratio, whether it is achieved by mixing and heating the mixture until a transparent liquid is formed, dissolving components in water and subsequent evaporation or freeze-drying, or even by grinding [157]. Many researchers have recognized their potential for various applications, and DESs have been extensively used in the synthesis of heterocyclic compounds [165,166,167], biomass valorization [168,169,170], the extraction of bioactive compounds [171,172,173], and electrodeposition [174,175,176,177]. 

## 4. Extraction of Tannins Using Deep Eutectic Solvents

As deep eutectic solvents have been found to be very effective in many extraction processes, in recent years, authors have used them as a green alternative to conventional solvents in the extraction of different bioactive compounds, including tannins, from plants or plant-based by-products derived from different production processes. Data in the literature on the use of DESs in tannin-based compounds is still scarce compared to other classes of compounds, but some general conclusions can be drawn. Additionally, only a few classes of plant-based materials have been investigated, with the most abundant data obtained for chestnut wood or trunk, pomegranate peels, grape pomace, and onion peels. 

Chestnut wood and trunk are rich in tannins, and different tannins, such as castalin, vescalin, castalagin, and vescalagin, can be extracted from this plant material [6]. During food production, chestnut shell is usually disposed of as waste; however, it could be used as an excellent source of different bioactive compounds [178,179]. Conventional extraction of these compounds from chestnut processing waste involves the use of water [6,180], 50% ethanol [181], subcritical water [182], and 1% Na_2_SO_3_ or 1% NaOH [183]. DESs could be effectively applied in this manner, maintaining the green character of the process and demonstrating superior performance compared to other solvents, as evidenced by the research of some authors described as follows. It has been found that proanthocyanidins could be effectively extracted from chestnut shell using choline chloride:oxalic acid dihydrate DES [179]. Husanu et al. (2020) compared the extraction efficacy of various DESs with conventional solvents in obtaining polyphenols and proanthocyanidins. After extraction with DES, the extracts were treated with polymeric resin to separate the extracted compounds. Acid-based DESs proved more effective in extracting proanthocyanidins (as determined by a vanillin–HCl assay) compared to those based on sugars (maltose and glucose) and polyols (ethylene glycol and 1,4-butanediol), resulting in extracts of different colors, which indicated variations in extract composition. DES extraction was also compared to conventional extraction using methanol/water as a solvent with the same extraction parameters. While choline chloride:oxalic acid dihydrate DES extract showed a yield of 189.6 mg_CE_/g_BM_ (CE, catechin hydrate equivalents; BM, biomass), the methanol/water condensed tannin yield was 39.03 mg_CE_/g_BM_, showing the superior extraction efficacy of the mentioned DES. Keeping in mind the green character of the process, it is important to mention that DESs could be recycled and reused for fresh biomass waste extraction [179]. This capability significantly enhances the sustainability of the method, as it reduces the need for fresh solvents, thus minimizing waste and lowering the environmental footprint associated with the extraction of valuable compounds from fresh biomass waste. In continuation of this research, Gonzales-Rivera et al. investigated the use of the above mentioned acid-based DESs, with addition of levulinic and oxalic acid-based DESs, in combination with microwaves for extraction of polyphenols and total proanthocyanidins from chestnut shell [178]. Polyphenols were separated from the extract using the same resin as before, Amberlite XAD-7. Among the DESs used, the most effective one was found to be the choline chloride:oxalic acid dihydrate, which according to their investigation, showed a high microwave heating response as compared to choline chloride:oxalic acid DESs or the compound with the water added to the DES during or after its formation. This shows that not only the presence but also the nature of water has a huge impact on DES properties, as well as the overall extraction process. In addition, if compared to the previous research described above [179], it can be concluded that microwaves promote this extraction to obtain higher tannin yield (229.6 mg_CE_/g vs. 189.6 mg_CE_/g) in a shorter extraction time (60 min vs. 24 h). The authors found it difficult to handle some of the DESs used due to their high viscosity, which is usually described as one of their main disadvantages of these compounds, and could be overcome to some extent by the addition of water. Furthermore, compounds extracted using DESs could not be recovered by evaporation of the solvent as in conventional extraction, due to the low vapor pressure of DESs. Therefore, the recovery of extracted compounds is usually performed using various resins, as in previously mentioned research, which complicates, prolongs, and raises the price of the overall process. Aside from the aforementioned results, this research showed that DESs in combination with microwaves are very effective in delignification of chestnut shells [178]. Considering lignin extraction from chestnut wood, Moccia et al. [184] used chestnut wood fiber to obtain extracts rich in ellagic acid and lignin. They optimized methods to obtain either ellagic acid-rich extracts or lignin-rich extracts, thus using the tuning capacity of DESs, which is one of their most prominent features. When a choline chloride:tartaric acid DES was used in the first extraction step, ellagic acid was obtained, while subsequent extraction of the residual material using choline chloride:lactic acid DES yielded lignin-rich extracts. Once again, an acid-based DES found its application in the delignification of plant-based material, as in many other studies dealing with this subject [185]. 

A profound group of tannins, proanthocyanidins, are known for their biological activities and are found in many different plants as secondary metabolites [20,186,187]. *Ginkgo biloba* L., a widely used medicinal plant, contains proanthocyanidines, among other compounds [188]. The extraction of tannins from different materials is usually performed using conventional solvents [189,190,191], but Cao et al. proved that DESs could be even more effective. They compared the extraction efficacy of choline chloride-based DESs, which were combined with different alcohols, acids, or urea as HBDs. Acid-based DESs were found to be the most efficient, and the authors assumed this could be due to their higher polarity compared to other DESs. The authors also prepared betaine-based DESs, but their potency to extract proanthocyanidines was lower than the choline chloride-based DESs. The authors speculated that this could be due to stronger hydrogen bond interactions between chloride ions and proanthocyanidines. Finding the viscosity of DESs is one of the major limitations in the extraction process. When water is added to a DES, decreasing its viscosity and surface tension, it also changes the polarity of the solvent. The addition of water increases the yield of extracted proanthocyanidines to some extent, after which water weakens hydrogen bonding, having a negative impact on proanthocyanidine yield. In one study, RSM resulted in optimal extraction conditions (listed in Table 2), predicting a yield of 22.23 mg/g, while the experiment yielded 22.10 mg/g. They also compared the extraction yields of proanthocyanidins using conventional solvents like 70% methanol, 70% ethanol, and 70% acetone with a choline chloride:malonic acid:H_2_O DES under the same (optimal) conditions. The proanthocyanidin yield obtained using DESs was higher than with any of the conventional solvents (70% methanol 7.87 mg/g, 70% ethanol 7.84 mg/g, and 70% acetone 13.26 mg/g) [188]. RSM in general has been found to be an excellent tool in the optimization of process parameters and is often applied in extraction processes. Although it can provide optimal process parameters, which are usually in accordance with experimental ones, it cannot be used as a tool to investigate and describe interactions between solvents and compounds of interest. In this manner, some authors have applied molecular dynamics simulation studies, which provide a deeper insight in solvent–compound interactions, contributing to a better understanding of the superior efficacy of DESs in the extraction of some compounds. This tool was used in the investigation of proanthocyanidins’ extraction from cottonseed hulls, another industry by-product, by Cui et al. They combined the use of DESs with ultrasonication to extract proanthocyanidines using 14 different DESs. The most effective DES among the 14 of them was choline chloride:levulinic acid, which was used for further optimization processes in RSM. The optimization analysis revealed the optimal parameters listed in Table 2, with an expected yield of 78.58 mg/g cottonseed hulls, while the actual yield was 75.25 mg/g. Closer examination of intermolecular interactions by molecular dynamics was performed for catechin and a DES in comparison to other solvents (methanol and water), revealing that the attractions between the DES and catechin were stronger than to other solvents, influencing the extraction yield. This could probably be applied for other compounds as well, where DES has shown better extraction efficiency. Comparison to conventional solvents, including 70% methanol, 70% ethanol, and 70% acetone, once again showed that the above-mentioned DES is more suitable for this extraction. The yields obtained by conventional solvents were lower than the one obtained with DES, as follows: 70% methanol 36.44 mg/g, 70% ethanol 30.87 mg/g, and 70% acetone 43.41 mg/g, with all the extractions performed under sonication [186]. 

Pomegranate (*Punica granatum* L.), a fruit often cultivated in subtropical and tropical parts of the world, is known for its beneficial health effects, mostly due to the presence of different phytochemicals. The content of hydrolysable tannins in pomegranate depends on the fruit part, variety, development stage, and climate [192]. Pomegranate peels are often discarded after fruit processing, thus becoming a by-product rich in bioactive compounds, including tannins. In their attempt to valorize this kind of waste, Rajha et al. combined ultrasonic and IR technology with DESs in the extraction of polyphenols and tannins from pomegranate peel [193]. They found that the DES’ extraction efficiency depended on the target compounds, with the most efficient DES for tannin extraction being choline chloride:fructose DES (in combination with ultrasound), with very similar results obtained for the combination of IR and choline chloride:lactic acid DES. The choline chloride:lactic acid DES was efficient in the extraction of both polyphenols and tannins, while the choline chloride:fructose DES was suitable for extraction of tannins only. They also compared their results with conventional solvent extraction, using water and an ethanol/water mixture. While the tannin yield obtained by both of the above-mentioned DESs was ≈ 50 mg/g, yields obtained using water and ethanol/water were ≈15 mg/g and ≈25 mg/g, respectively [193]. Hernandez-Corroto et al. also investigated the utilization of DESs in the extraction of different polyphenols, including ellagitannins, from pomegranate by-products, using discarded seed. The first step prior to extraction was high-voltage electrical discharge (HVED) pretreatment, causing cell disintegration. Five different DESs were investigated, including three acid-based formulations and others with the addition of glycerol and glucose. They investigated the polyphenol yield, with most of the extracted polyphenols identified as ellagitannins. The investigation revealed that among the tested DESs, the diffusivity coefficient was higher for acid-based DESs. However, the highest was observed in an alkaline solution using NaOH. Similarly, the yield of total polyphenols was greater for the alkaline solution than for any of the DESs tested [194]. 

Grape pomace, a wine production by-product, is rich in proantocyanidins. Many agro-industrial residues rich in tannins, like grape skins and seeds discarded after wine production, are being processed to extract tannins, which can then be used in various applications, thereby reducing waste and contributing to a more sustainable industry cycle. Neto et al. investigated the possibility of changing the white grape pomace extract composition by varying the DES component ratio, and more precisely the ratio of water and ethanol to pure DES. They found that DES composition and extraction time and temperature influence not only the proanthocyanidin yield, but the mean degree of polymerization (mDP) and galloylation percentage as well. Among the investigated DESs (choline chloride, betaine, and proline-based), choline chloride-based DESs with 25% H_2_O or 25% H_2_O/ethanol showed superior efficacy (80.7 mg_PAC_/g_BM_ for choline chloride:urea and 81.4 mg_PAC_/g_BM_ for choline chloride:malic acid; PAC, proanthocyanidins) considering proanthocyanidine extraction. However, due to stability issues with choline chloride:malic acid DESs, especially when exposed to high temperatures, as well as well as the low mDP values obtained with the choline chloride:urea DES, the authors chose the choline chloride:glycerol:water:ethanol DES for further optimization. They found that glycerol had an adverse effect on proanthocyanidine extraction, thus taking into consideration the solvent formed of choline chloride, water, and ethanol. Considering the highest proanthocyanidine yield of 143.0 mg_PAC_/g_BM_ (predicted by model), the optimal conditions would be 14.4% of biomass, a temperature of 102.8 °C, time of 5 h, and an experimental value of 144.1 mg_PAC_/g_BM_. However, since other parameters the authors investigated were obtained in low values, another model was developed to compensate for the desired outcomes. Therefore, with a temperature of 99 °C, 13.4% of biomass, and an extraction time of 1 h, the predicted proanthocyanidine yield was 133.6 mg_PAC_/g_BM_, agreeing well with the experimental yield of 125.9 mg_PAC_/g_BM_. They also investigated the efficiency of conventional solvents, such as hot water, hot sulfite solution, acetone/water, and ethanol/water, in the extraction of proanthocyanidines, obtaining the highest yield of 94.4 mg_PAC_/g_BM_ with the acetone/water mixture (70% *v*/*v*) over 4 h at 30 °C. As the authors stated, when considering solvent removal, acetone is still the better option for extraction [187]. In their later research, they investigated the influence of microwaves on the extraction process of proanthocyanidines from grape pomace [195]. They studied proanthocyanidin stability in different DESs, noticing that their degradation was highly influenced by the DES type and temperature. The lowest degradation was observed in DESs where choline chloride was mixed with glucose and glycerol. In addition, they found that some DESs are prone to thermal degradation when exposed to microwaves, and that choline chloride-based DESs were the most stable, except when glucose was used as HBD. Furthermore, it is well known that in some acidic DESs where choline chloride is combined with carboxylic acids, esterification can occur over prolonged times at room temperature, and much faster at elevated temperatures [195,196]. In one study, choline chloride-based DESs were found to be more effective in proanthocyanidin extraction than DESs with betaine and proline as HBA. Choline chloride:lactic acid DESs were found to yield the highest proanthocyanidin content of 135.0 mg_PAC_/g_GP_ (GP-grape pomace) and had the highest mean degree of polymerization, and the process was optimized using this DES, yielding process parameters as described in Table 2. The combination of MAE with choline chloride:lactic acid DESs under optimized conditions provided an efficient, environmentally friendly, and time-consuming method for proanthocyanidin extraction [195].

Tea leaves can also be a good source of tannins, and they can be extracted using a glycerol:acetic acid DES in combination with ultrasound technology, as well as via enzymatic-assisted extraction [197,198]. Screening of different NADESs to obtain the highest total tannin content (TTAC) revealed that the acetic acid:glycerol:water DES was the most efficient, being superior to ethanol as well. When DES extraction was performed under ultrasonication conditions, increasing the power up to 750 W increased the tannin yield. Under further increases, the TTAC was lowered, which authors explained using the saturation effect, meaning that the destructive effect on plant material was diminished by bubble coalescence. Temperature also had a significant effect on TTAC, with 60 °C showing the best impact, while at higher temperatures, degradation of tannins probably occurred. The water content of DESs also increased TTAC to some extent, with the optimal portion of water being 20%. In addition to these parameters, the authors found that this extraction could be enhanced in combination with enzymatic hydrolysis of cell walls. Here, one must be cautious with the pH of the media, which in this case was regulated by the addition of a specific amount of sodium acetate. This combination was found to be the most effective, exposing the plant material to ultrasound with subsequent enzymatic treatment, yielding TTAC of 197.0 mg_CE_/g_BM_ [198]. 

NADES were also found to be an excellent media for conversion of ellagitannins to ellagic acid, with subsequent extraction of ellagic acid (EA), from raspberry seed [199]. Teslić et al. investigated DESs formed of HBAs such as fructose, glucose, or betaine in combination with different acids, such as HBDs (lactic acid, malic acid, tartaric acid, citric acid), with a water content of 20–25%. The citric acid:betaine:water DES yielded the highest amount of EA (75.17 mg/100 g_DW_; DW, dry weight) among all of the DESs used, as well as compared to 80% ethanol, under the same conditions. Obviously, DESs induce better conversion of ellagitannins to ellagic acid, which is understandable since acidic conditions favor this process. On the other hand, the use of methanolic 2M HCl, a strong acid, induced better ellagitannin conversion to ellagic acid than the DESs, yielding 1031.41 mg/100 g_DW_ of EA at 85 °C over the course of 150 min. Authors also tried to combine ultrasound with DES extraction, but this attempt yielded lower EA content [199]. 

The consumption and processing of onion, as one of the major world crops, yields large amounts of onion peel, which is a valuable by-product rich in bioactive compounds [200]. The valorization of onion peels could be achieved by the extraction of those bioactive compounds, such as anthocyanins, tannins, flavonoids, and other compounds. So far, conventional methods have mostly been applied, but recently, ultrasound and microwave-assisted subcritical water extraction and supercritical fluid extraction have found their effective application in these extractions [201]. DESs are also being applied, as researchers have recognized their advantages over conventional solvents [202,203,204,205]. The most common onion peel tannins found by Sukor et al. were pedunculagin, tellimagrandin, 3,4,5-*O*-tri-caffeoyl-quinic acid, norbergenin, and tannic acid. They applied DESs for onion peel extraction in combination with ultrasound, investigating the influence of process parameters on the yield of tannic acid, while comparing it to methanol extraction. Their results showed that the applied DES was much more effective in tannic acid extraction than methanol when the same parameters were applied (641.16 µ/g vs. 368.99 µg/g) [206]. Further optimization led to even higher tannic acid yield [207]. Most of the previously mentioned research has investigated different types of DESs in the extraction of desired compounds, while here, the authors used one type of DES, changing only the component ratio. Choline chloride and urea were mixed in 1:1, 1:2, and 2:1 ratios, affecting the physicochemical properties of the DES, with each showing a different extracting capacity for tannic acid. The most efficient component ratio was found to be 1:1 with a solid to liquid ratio of 10:1, providing a tannic acid yield of 1705.8 µg/g. The authors pointed out that the higher choline chloride content increased the DES’ surface tension, while higher urea content caused higher steric hindrance and weakened the interaction of the solvent with target compounds. Ultrasonication was found to have great impact on cell tissue damage as seen by SEM imaging, which enabled better diffusivity of tannic acid from plant material [206]. 

Yellow cytinus (*Cytinus hypocistis* L.) is a plant used in traditional medicine, due to its potential health benefits. In traditional medicine, it is used for dysentery treatment, to reduce inflammation, and for healing scars, and it is known for its high content of tannins [207]. Zengin et al. compared different solvents, both conventional (hexane, ethyl acetate, dichloromethane, ethanol, ethanol/water, and water) and NADESs (choline chloride:urea, L-proline:xylitol protonated with HCl, and L-proline:xylitol protonated with H_2_SO_4_) for extraction of bioactive compounds from *Cytinus hypocistis* (L.). Among 148 compounds identified, 61 of them were gallotannins and 30 were ellagitannins. Specific compounds were identified, but not quantified themselves; however, total phenolic content (TPC) and total flavonoid content were determined. TPC was the highest for NADES extracts, with L-proline:xylitol protonated with HCl being the best at 186.13 mg GAE/g. Conventional solvents yielded lower TPC than NADES, with ethyl acetate being the most efficient among them [207].

A special group of tannins, phlorotannins, which are found in brown algae, are formed of phloroglucinol units. They also show a wide spectra of biological activities, which makes them an excellent substrate for extraction [208,209], especially given that they are allowed for use as dietary supplements in the EU [210]. Their extraction from different algae can be performed by different DESs, as was shown by Obluchinskaya et al. [209]. They performed phlorotannin extraction from two brown algae (*Fucus vesiculosus* L. and *Ascophyllum nodosum* (L.) Le Jolis) using natural deep eutectic solvents (NADES) composed of two or three components in different ratios, using choline chloride, glucose, and betaine in combination with malic acid, lactic acid, glycerine, and water. The betaine:lactic acid:water (1:1:2) DES showed the highest yield of extracted phlorotannins (61.2 mg/g_DW_ for *F. vesiculosus* and 50.3 mg/g_DW_ for *A. nodosum,* comprising 34–35% of the total phlorotannin content). Furthermore, they proved that the extraction efficacy of selected NADES could be improved by the addition of water, where the extent of improvement depended on the NADES itself. The choline chloride:lactic acid NADES showed the highest extracting capacity when water was added, as well as the highest increase in this capacity by the addition of water. Compared to the pure NADES, their efficiency was tenfold better, whereas the betaine:lactic acid:water NADES capacity was not significantly improved with the addition of water. This proves that many solvent-related factors affect the extraction capacity, solvent polarity, viscosity, and the ability of the solvent to interact with desired compounds, all depending on the DES’ components, their ratio, and the addition of water [209]. In their further research, they investigated the combination of NADES and ultrasonication in the extraction of phlorotannins from *Fucus vesiculosus* L. [211]. Choline chloride:lactic acid (1:3) and lactic acid:glucose:water (5:1:3) NADESs were investigated and compared to ethanol extraction. The phlorotannin yield obtained with both NADES (17.2 and 9.3 mg/g seaweed) was lower than the yield obtained with ethanol (71.7 mg/g seaweed). The efficacy of the mentioned NADES in extracting phlorotannins was significantly improved by the addition of water (up to 30%) and prolongation of the extraction time (to 60 min). UAE combined with NADES, performed for 60 min at 25 °C, was the method of choice for recovering phlorotannins and other lipophilic and hydrophilic compounds from the aforementioned algae. In addition, they investigated the stability of phorotannins in both NADES (with addition of 30% H_2_O) and ethanol during certain periods of time. Results showed that NADESs can enhance compounds’ stability compared to conventional solvents, which after 360 days was the highest in choline chloride:lactic acid, followed by lactic acid:glucose:water, and the lowest was in ethanol [211]. In their subsequent research, they further investigated and optimized conditions for UAE phlorotannin extraction using only a choline chloride:lactic acid (1:3) DES. They optimized the process parameters, obtaining results for a water content of 30%, the same as in their previous research, a time of 22.8 min, a solid:liquid ratio of 1:12, and with predicted yield of 140.3 mgPGE/g_DW_, which was in accordance with their obtained yield of 137.3 mgPGE/g_DW_ (PGE-Phloroglucinol Equivalents). This was a continuation of their research mentioned previously, showing that in this case, a much higher phlorotannin yield was obtained, proving the importance of optimization processes [212]. 

This section summarizes the use of DESs in the extraction of tannin compounds from different plant-based materials. Although different materials were examined, and structurally different tannins were extracted, a general conclusion arises from most of the reviewed data. In most cases, the most effective solvents were acid-based DESs. Not only were acid-based DESs the most effective among the different DESs examined, but they also showed superior efficacy compared to conventional solvents. This increased effectiveness could be attributed to stronger interactions between these DESs and the desired compounds than those formed by conventional solvents. Many researchers have explored the tunability of the DESs in order to improve the extraction of desired compounds, and based on optimization results, authors chose the best extraction parameters. The overall impression is that more environmentally friendly solvents, such as DESs, can replace conventional ones, being even more effective in extraction of tannins. Nevertheless, this opens many new paths and directions for investigation and application of DESs to obtain desirable outcomes. 

**Table 2:** Extraction conditions of different tannins from plant material using DESs.

Compounds	Yield	Plant Material	Parameters	Reference
Total proanthocyanidins	189.6 mg_CE_/g_DW_	Chestnut shell	5 g of DES:0.5 g of CSW, 65 °C, 24 h, Amberlite XAD-7	[179]
Proanthocyanidins	229.6 mg_CE_/g_BM_	Chestnut shell	Choline chloride:oxalic acid dihydrate, 1:10 (solid:liquid), MW, 60 min, 85 °C, Amberlite XAD-7	[178]
Hydrolysable tannins		*Alchemilla vulgaris* L.	Choline chloride:urea (1:2), 50% water, 68.2 min, 30 °C	[213]
Proanthocyanidins	22.10 mg/g	Gingko biloba leaves	Choline chloride:malonic acid (1:2), 55% H_2_O, 65 °C, 53 min, 10.57:1 (*V*/*w*), macroporous resin D-101	[188]
Proanthocyanidins	75.25 mg/g	Cottonseed hulls	Choline chloride:levulinic acid (1:2), UAE, 33.21% water, 36.25 mL/g (liquid:solid ratio), 7.40 min	[186]
Ellagitannins		Pomegranate seed	HVED preatreatment, Choline chloride:citric aicd/acetic acid/lactic acid, 50 °C, 1:10 (liquid:solid), 60 min, 160 rpm	[194]
Tannins	50 mg/g_DW_	Pomegranate peel	US, choline chloride:fructose, 1:10 (solid:liquid), 50 °C, 90 min	[193]
Proanthocyanidins	144.1 mg_PAC_/g_BM_	Grape pomace	Choline chloride:ethanol:water, 14.4% biomass, 102.8 °C, 5 h	[187]
Proanthocyanidins	135 mg/g	Grape pomace	MAE, choline chloride:lactic acid:water (0.36:0.39:0.25), 99.2 °C, 3.56 min	[195]
Phlorotannins		Brown algae (*Fucus vesiculosus* L. and *Ascophyllum nodosum* (L.) Le Jolis)	Choline chloride:lactic acid, 20% H_2_O, maceration, 2h, 50 °C	[209]
Phlorotannins		*Fucus vesiculosus*	UAE, lactic acid:choline chloride or lactic acid:glucose:H_2_O, 25 °C, 60 min, 1:10 (solid:liquid)	[211]
Phlorotannins	137.3 mg_PGE_/g_DW_	*Fucus vesiculosus*	UAE, choline chloride:lactic acid, 23 min, 30% water, 1:12 (solid:liquid)	[212]
Tannic acid	1705.79 µg/g	Onion peel	UAE, Choline chloride:urea (1:1), H_2_O, 1:10 (solid:liquid), duty cycle of 10%	[206]
Ellagic acid	5.21 mg/100 g_extract_	Raspberry seed	Citric acid:betaine:H_2_O (2:1:2), 85 °C, 147 min, 1:15.76 (solid:liquid)	[199]

CE: catechin hydrate equivalents, PAC: proanthocyanidins, PGE: phloroglucinol equivalents, DW: dry weight, BM: biomass.

## 5. Biological Activity of Plant Tannins

During the last few decades, the biological effects of tannins have been extensively studied using various in vitro or animal models [214] (Table 3). According to research, tannins can show their biological activity as indigestible and digestible tannins and metabolites produced by the fermentation of tannins. Tannins and their complexes show different effects, such as antioxidant, radical scavenging, antimicrobial, antiviral, anticancer, and antinutrient activity [20,215,216,217,218,219].

Accumulation of reactive oxygen species (ROS) in cells is usually associated with inflammatory processes in the body, leading to chronic degenerative diseases such as cardiovascular diseases and diabetes mellitus and neurodegenerative diseases such as Parkinson’s and Alzheimer’s disease [251]. Natural bioactive components, due to their mode of action, could offer a solution in combating oxidative processes. Research indicates that tannins exhibit antioxidant activity through the inhibition of lipid peroxidation, scavenging of free radicals, and chelation of transition metals, as well as the mediation and inhibition of enzymes [252]. Tannins enhance ROS detoxification in plant cells by modulating antioxidant enzymes like superoxide dismutase and catalase [253], and by synergistically interacting with non-enzymatic antioxidants such as ascorbic acid and tocopherols [254]. They also stabilize cellular membranes, reducing fluidity and permeability under oxidative stress [255]. Their antioxidant activity is related to their composition and polymerization [214]. Salah et al. found that the strength of free radical inhibition increased with the quantity of phenolic hydroxyl groups and the degree of polymerization of tannins [233]. Twenty-three of the twenty-five distinct types of tannins and other pertinent compounds that Okuda et al. investigated showed varying degrees of antioxidant activity based on the location and quantity of phenolic hydroxyl groups [237]. The process of lipid peroxidation is also inhibited by the use of hydrolysable tannins and proanthocyanidins. Hydrolysable tannins, especially pedunculagin, pentagalloylglucose, and geraniin, demonstrate strong inhibition of lipid peroxidation in animal cells [214]. Extracts containing proanthocyanidins, notably procyanidin and prodelphidin units, also exhibit antioxidant activity. Based on the behavior of extracts with different radicals, extracts containing proanthocyanidins are considered to be radical scavengers [256].

Tannins have significant anti-inflammatory and wound-healing properties. Tannins from seedling leaf tissue and callus culture extracts of *Achyranthes aspera* L. and *Ocimum basilicum* L. were found to have anti-inflammatory properties [257]. According to some recent research, tannins reduce inflammation by blocking pro-inflammatory prostaglandin-E2 (PGE2) and NO [258]; however, the exact mechanisms can vary depending on the type of tannin and the specific biological context. Grape seed procyanidin extract was used in an in vitro experiment with obese Zucker rats to establish its ability to decrease inflammation triggered by obesity through altering the expression of cytokines [259]. Further research is needed to fully elucidate the molecular mechanisms underlying the anti-inflammatory effects of tannins.

The alarming rise in the prevalence of antibiotic-resistant bacteria, posing challenges in infection treatment, has triggered the search for new antibacterial compounds and alternative strategies to combat bacterial infections. Bioactive components of plant origin may exhibit direct antibacterial activity and/or indirect activity as compounds by modifying antibiotic resistance, thereby enhancing antibiotic efficacy when used in combination. These bioactive components possess the capability to alter or inhibit resistance mechanisms, rendering bacteria susceptible to antibiotics or enabling antibiotics to function at lower concentrations. A systematic investigation of plant-derived bioactive compounds, particularly those capable of synergizing with antibiotics as resistance-modifying agents, represents a potential strategy to overcome bacterial resistance [260]. Tannins belonging to the group of phenolic compounds exhibit antimicrobial properties, with an emphasis on antibacterial activity. Antibacterial activity in the context of inhibiting the growth of potentially pathogenic antibacterial strains is also shown by hydrolysable tannins and proanthocyanidins, while also having no negative effect on positive gut bacteria [261]. Additionally, tannins are frequently utilized as antimicrobial agents in animals, considering that they can inhibit extracellular microbial enzymes and reduce the supply of substrates and essential minerals to microbial organisms [262]. The mechanisms underlying the antibacterial action of tannins involve the chelation of iron, the inhibition of cell wall synthesis, and the modulation of the cell membrane, particularly affecting membrane potential and permeability [218]. The antibacterial activity of tannins is associated with their structure, especially with the hydroxyl groups they contain. Research indicates that gallotannins exhibit higher antibacterial activity compared to ellagitannins [218]. Tannic acid, being one of the most extensively studied gallotannins, has demonstrated potent antibacterial activity against a wide range of tested bacteria, which confirms the superiority of gallotannins [120,218,230,263,264]. Extracts from two Mediterranean species of the plant *Cytinus* containing gallotannins, in particular 1-*O*-galloyl-β-d-glucose and pentagalloyl-*O*-β-d-glucose, showed significant antibacterial activity against *S. aureus*, *S. epidermidis*, *P. aeruginosa*, *K. pneumoniae*, and *E. faecium*. Experimental studies with synthetic components suggested that pentagalloyl-*O*-β-d-glucose is the active antimicrobial component of the extract [221]. Punicalagin and corilagin, as representatives of ellagitannins, also possess antibacterial properties, particularly against *S. aureus* and *H. pylori*. Punicalagin, in particular, demonstrates the potential to suppress the resistance of *S. aureus* to the antibiotic oxacillin, indicating synergistic action with the antibiotic [238]. Additionally, vescalagin, castalagin, vescalin, and castalin, representatives of ellagitannins obtained from crude chestnut extract, demonstrate better antibacterial activity against *E. coli* compared to *S. aureus*, suggesting a varying action of tannins based on their structural differences [247]. Procyanidin and prodelphinidin, as proanthocyanidins, demonstrate good antibacterial activity, particularly against gram-positive bacteria associated with skin diseases, such as *Staphylococcus epidermidis*, *Salmonella enterica ser. choleraesuis, Proteus vulgaris*, *Enterococcus faecium*, *Bacillus subtilis*, *Acinetobacter caicoaceticus var. anitratus*, and *Staphylococcus aureus*. The problem with these bacteria is that they often develop resistance to existing commercial antibiotics [256], which could be avoided by using tannins alone or in combination with antibiotics. Proanthocyanidins, especially prodelphinidins, exhibit strong antibacterial activity against pathogenic *E. coli*, affecting swarming motility and inhibiting biofilm formation. Since tannins operate through mechanisms distinct from those of existing antibiotics, there is a belief that resistance to tannins is less likely to develop [265]. Considering the comprehensive range of antibacterial activities demonstrated by tannins, they represent a promising alternative or complement to conventional antibiotics. This aspect makes them particularly interesting for applications in the food, medical, and pharmaceutical industries.

In addition to antioxidant and antibacterial assessments, research on the impact of natural bioactive components on cancer cells is prevalent. The influence of tannins on the process of cancer formation has been monitored for the last 30 years, resulting in a comprehensive understanding of the molecular targets of tannins. Mechanisms of the anticancer action of tannins include the modulation of signaling pathways in cancer cells, the induction of apoptosis and autophagy, and the reduction of cancer stem cell population by downregulation of the expression of specific biomarkers, such as CD44 and ALDH1 [266,267]. The influence of tannin through these mechanisms has been mostly studied with compounds such as ellagic acid, tannic acid, and epigallocatechin gallate. For instance, tannic acid demonstrates a positive effect on the signaling pathways involved in cancer cell regulation, as observed in human embryonic carcinoma cells and in lung cancer A549 cells [231]. Tannic acid also shows an effect by inducing apoptosis in human liver hepatocellular carcinoma HepG2 cells and gingival squamous cell carcinoma. An additional benefit of tannic acid in this process is its capability to remove iron from cells in the form of Fe^3+^–TA complexes, whereby the occurrence of cancer or diseases associated with excess iron can be prevented [268]. Epigallocatechin gallate, as a flavan-3-ol building block of proanthocyanidins, affects various signaling pathways, leading to destabilization and subsequent activation of apoptosis in renal cell carcinoma, human neuroblastoma cancer cells [269], and colorectal cancer cells [270]. Additionally, epigallocatechin gallate suppresses cancer cell growth and induces apoptosis in hepatocellular carcinoma HepG2 cells and human thyroid carcinoma (TT, TPC-1, and ARO cell lines) [271]. Ellagic acid, which is formed by the hydrolysis of ellagitannins in the body during digestion, influences cell signaling to trigger apoptosis of various cancer cells, including human bladder cancer T24 cells [272], melanoma cell lines (205Lu, WM852c and A375) [273], and colorectal cancer HCT116 cells [274]. The ellagitannins isolated from *Acer pseudosieboldianum* (Pax) Komarov leaves exhibit potential for inducing cancer cell apoptosis and chemopreventive activity on prostate cancer cells. Among the tannins investigated for potential prostate cancer therapy, geraniin, granatin, mallotusinic acid, and komaninin have demonstrated notable effectiveness [224]. The influence of proanthocyanidins in the extract, with emphasis on procyanidins and prodelphidins, was tested on human breast adenocarcinoma (MCF-7), human colon carcinoma (HT29), human cervical carcinoma (HeLa), and human liver carcinoma (HepG2) cell lines. The best effect was observed in MCF-7 cell lines, with an IC_50_ value of 38.33 ± 2.08 μg/mL. Furthermore, proanthocyanidins induced apoptosis in MCF-7, Hep G2, and HT29 cancer cells, underscoring their potential anticancer activity and highlighting the need for further research in this area [256].

The utilization of chemotherapeutic drugs is often associated with the development of tumor resistance, as well as toxicity towards healthy cells during the treatment period. Numerous studies have demonstrated that tannins, through modulation of various signaling pathways, can reduce the occurrence of tumor resistance to drugs and minimize the adverse effects of drugs on healthy cells [275,276]. Combining chemotherapy with tannins may potentially enable the use of a lower required dose of drugs or radiation to achieve an equivalent treatment effect [276]. This suggests a promising avenue for enhancing the efficacy of, and reducing the side effects of, cancer therapy.

Current clinical trials are investigating the anticancer effects of tannins and tannin building blocks, and include trials of epigallocatechin gallate, either administered alone or more commonly in the form of green tea or extract. When using tea or extracts containing tannins, alongside numerous other bioactive components, isolating the specific effect of tannins becomes challenging. In such instances, any observed positive effects are often attributed to the synergy among the various components present [266]. This underscores the complexity of natural products and highlights the importance of considering interactions among multiple compounds in evaluating their therapeutic potential. Tannin-rich extracts are incorporated into dietary supplements, teas, and fortified foods, aiding in managing cholesterol levels, improving cardiovascular health, and reducing cancer risk [252].

## 6. Tannin-Based DESs

It is interesting to mention that some researchers have developed new deep eutectic solvents using tannins. Zhu et al. prepared polymerizable ternary DESs formed of tannic acid, choline chloride, and hydroxyethyl methacrylate. They investigated the formation of hydrogen bonds and van der Waals forces between the components of the DESs and their physical and chemical properties, suggesting their potential use in 3D printing processes [277]. Picchio et al. also prepared a DES using tannic acid. They combined it with choline chloride in a 20:1 choline chloride:tannic acid ratio to obtain a viscous brown liquid. This DES was characterized using different methods, indicating its high thermal stability, with the ability to act as a corrosion protector for mild steel when combined with a polymer coating [278]. Lacalle et al. also prepared a DES formed of tannic acid, but in this case the HBAs were [2-(methacryloyloxy) ethyl] trimethylammonium chloride or [2-(acryloyloxy) ethyl] trimethylammonium chloride, in an HBA:HBD ratio of 20:1. In both cases, a viscous brownish liquid was obtained [279]. These materials showed high *T*_g_ values, and due to multiple hydrogen bonds formed within the solvent, they could not be deformed or could endure only small deformations easily and were brittle. Additionally, a methacrylic tannic acid-based DES was shown to have great Fe^3+^ complexation activity [279].

## 7. Conclusions

Tannins are a group of compounds with significant biological potential, and their extraction from different plant material can be performed using various techniques. With the current emphasis on minimizing the environmental impact of chemical processes, there has been growth in the use of green extraction methods and solvents. DESs comply with the green chemistry concept, and they have been successfully applied in the extraction of tannins from plant material. The possibility of tuning their properties makes them adjustable for specific uses and for application in obtaining extracts with desirable content levels of different compounds. In this manner, DESs have been found to be very efficient in tannin extraction, and in some cases, have been shown to be even better than conventional solvents. This research has noted various obstacles, such as the high viscosity of some DES formulations, which can be partially mitigated by adding water. Another detected obstacle was the intricacy of the recovery of compounds using DESs due to the low vapor pressure of such solvents, which precludes conventional evaporation techniques. Instead, recovery often requires the use of various resins, complicating and extending the process while increasing costs. Moreover, the study revealed that some DESs, especially those based on choline chloride, are susceptible to thermal degradation when exposed to microwave treatment. Finally, we recognized that esterification can occur in certain acidic DESs where choline chloride is mixed with carboxylic acids for an extended period of time at room temperature, and this process occurs considerably more quickly at higher temperatures. Although each plant material is specific and there exists a vast variety of structurally different tannins, a general conclusion based on the literature search could be that in most cases, acid-based DESs have shown a superior extraction capacity for tannins. Furthermore, research on this subject clearly indicates the tuning possibilities with regard to DES properties, making it important to optimize extraction conditions for each specific extraction setup. While DESs present certain operational challenges, their environmental and economic benefits, coupled with their superior extraction efficacy, especially when used in conjunction with other modern technologies like microwave heating, ultrasonic treatment, and enzymatic hydrolysis of cell walls, highlight their potential as sustainable alternatives to conventional extraction solvents. Given the presence of tannins in numerous plants and their diverse biological activities, there is considerable potential for further research on the application of DESs in tannin extraction. In the ongoing development of DESs for tannin extraction processes, some potential advancements could be implemented in future research, such as reducing viscosity without compromising solvation capabilities, developing new DES compositions, and minimizing the degradation of sensitive compounds. By focusing on these areas, the development of DESs can continue to progress towards environmentally friendly and economically viable solutions for industrial applications, particularly in the field of extraction technologies. As such, continued investigation into the application of DESs in tannin extraction promises to advance scientific understanding and practical applications across various fields. Moreover, this could open paths for the utilization of tannins across various industries, including pharmaceuticals, food, and cosmetics, unlocking new opportunities for product innovation and sustainable practices.

## Figures and Tables

**Table 3:** Activity of different tannins from plant material.

Compounds	Activity	Reference
**Hydrolysable Tannins**		
**Simple Gallic Acid Derivatives**		
1,2,3,4,6-penta-galloyl-d-glucopyranoside	Antibacterial activity against *Escherichia coli* (MIC = 32 μg/mL) and *Klebsiella pneumoniae* (MIC = 32 μg/mL)	[220]
	Antioxidant activity determined (TEAC-ABTS = 11.2 ± 0.8; FRAP = 8.4 ± 0.4; DPPH-scavenging = 6.2 ± 0.6; ORAC-PYR = 9.1 ± 1.4), results expressed as Trolox Equivalents (mM TE/g).	[221]
	Antioxidant activity by inhibiting superoxide anion radical in the hypoxanthine-xanthine oxidase system (IC_50_ = 3.4 μM)	[222]
	Antiviral activity against the COVID-19 by blockade the fusion of SARS-CoV-2 spike-RBD to ACE2 receptors (IC_50_ = 46.9 μM)	[223]
	Chemopreventive activity as anti-proliferative activity in androgen-independent prostate cancer PC-3 cells (IC_50_ = 242 µM) and in androgen-dependent LNCaP cells (IC_50_ = 0.81 µM)	[224]
Ginnalin B	Antioxidant activity by DPPH radical scavenging (IC_50_ = 12.14 µM), superoxide scavenging (IC_50_ = 20.80 µM) and inhibition of nitric oxide production (IC_50_ = 100 µM)	[225]
	Anti-inflammatory activity as α-glucosidase inhibition (IC_50_ = 38.5 µM)	[226]
Acertannin	Antioxidant activity by DPPH radical scavenging (IC_50_ = 6.87 µM), superoxide scavenging (IC_50_ = 2.96 µM) and inhibition of nitric oxide production (IC_50_ = 100 µM)	[225]
	Anti-inflammatory activity as *α*-glucosidase inhibition (IC_50_ = 88.42 µM)	[227]
Maplexin D	Antioxidant activity by DPPH radical scavenging (IC_50_ = 6.92 µM), superoxide scavenging (IC_50_ = 3.01 µM), and inhibition of nitric oxide production (IC_50_ = 100 µM)	[225]
Maplexin E	Antioxidant activity by DPPH radical scavenging (IC_50_ = 5.72 µM), superoxide scavenging (IC_50_ = 2.83 µM) and inhibition of nitric oxide production (IC_50_ = 36.08 µM)	[225]
	Anti-inflammatory activity as *α*-glucosidase inhibition (IC_50_ = 8.26 μM)	[227]
	Anti-inflammatory activity in rat paw oedema (35.3% of inhibition of oedema)	[228]
**Gallotannins**		
Tannic acid	Antibacterial activity against 50 methicillin-sensitive *S. aureus* and 50 methicillin-resistant *S. aureus*, (MIC from 40–160 μg/mL)	[229]
	Antibacterial activity against *Salmonella enterica* serovar Typhimurium, 40 µg/mL showed complete inhibition of bacterial growth	[230]
	Anticancer activity in non-small-cell lung carcinoma (NSCLC) category with no significant toxicity effects on human bronchial epithelial cells (IC_50_ = 40–60 μM at 24 h, 20–40 μM at 48 h)	[231]
	Anticancer activity in breast cancer MDA-MB-231 cells (IC_50_ = 2.5 μM) and in MCF-7 cells (IC_50_ = 4.0 μM)	[232]
	Anticancer activity in preventing liver cancer progression in vitro through inducing the mitochondrial-mediated apoptosis in HepG2 cells (IC_50_ = 360 μM)	[233]
	Anticancer activity in reducing cellular growth, clonogenic, invasive, and migratory capacities of pancreatic cancer cells C4-2 (IC_50_ = 2.92 μM), DU 145 (IC_50_ = 8.95 μM) and PC-3 cells (IC_50_ = 8.53 μM)	[234]
	Anticancer activity in gingival squamous cell carcinoma (GSCC) cellular proliferation in vitro (IC_50_ = 50 μM)	[235]
Octagalloylglucose	Anthelmintic activity tested in vitro against the egg hatching of *Haemonchus contortus*	[29]
**Ellagitannins**		
Pedunculagin	Antioxidant activity by DPPH radical scavenging (IC_50_ = 56 μM)	[236]
	Antioxidant activity by inhibiting superoxide anion radical in the hypoxanthine-xanthine oxidase system (IC_50_ = 2.8 μM)	[222]
	Most potent antioxidant activity by inhibiting lipid peroxidation in rat liver mitochondria and in rat liver microsomes (IC_50_ = 1.2 μM)	[237]
Punicalagin	Antibacterial activity against the six MRSA strains (MIC from 31.25–62.5 μg/mL)	[238]
	Antibacterial activity against *Staphylococcus aureus* (MIC = 250 μg/mL)	[239]
	Antibacterial activity against *Vibrio vulnificus* (MIC = 71 µg/mL)	[240]
	Antiviral effect as inhibitory effect on enveloped viruses known to use glycosaminoglycans for entry, including HCMV (IC_50_ = 16.76 µM), HCV (IC_50_ = 16.72 µM), DENV-2 (IC_50_ = 7.86 µM), MV (IC_50_ = 25.49 µM), and RSV (IC_50_ = 0.54 µM)	
Corilagin	Antibacterial activity against *Helicobacter pylori*, (MIC = 8 μg/mL)	[241]
	Antibacterial activity against *Mycobacterium smegmatis*, (MIC = 500 µg/mL)	[242]
	Weak antibacterial activities against *Bacillus subtilis*, *Staphylococcus aureus*, *Escherichia coli*, *Klebsiella pneumoniae*, *Pseudomonas aeruginosa* and *Proteus mirabilis* (MICs from 1000–2000 µg/ mL)	[243]
Chebulagic acid	Antimicrobial activity against plant pathogen *Erwinia carotovora* (19 mm), human pathogens *Staphylococcus aureus* (11 mm) and *Corynebacterium accolans* (10 mm) and human pathogenic yeast *Candida albicans* (12 mm), expressed as inhibition zone diameter (mm) for 100 µg of compound	[244]
	Anti-inflammatory activity by xanthine oxidase inhibition (IC_50_ = 48 µM)	[244]
	Weak antibacterial activity against multidrug-resistant *Acinetobacter baumannii* (MIC = 1000 µg/mL)	[245]
	Antibacterial activities against *Ralstonia solanacearum* and *Xanthomonas arboricola pv. pruni* (MIC = 52 μg/mL)	[246]
	Antiviral effect as inhibitory effect on enveloped viruses known to use glycosaminoglycans for entry, including HCMV (IC_50_ = 25 µM), HCV (IC_50_ = 12 µM), DENV-2 (IC_50_ = 13.11 µM), MV (IC_50_ = 34 µM), and RSV (IC_50_ = 0.38 µM)	
Geraniin	Antibacterial activity against 20 strains of *Staphylococcus aureus* (MIC = 190 µg/mL), 26 strains of the genus *Salmonella* (MIC = 1085 µg/mL), *Vibrio vulnificus* (MIC = 120 µg/mL)	[240]
	Chemopreventive activity as anti-proliferative activity in androgen-independent prostate cancer PC-3 cells (IC_50_ = 271 µM) and in androgen-dependent LNCaP cells (IC_50_ = 0.36 µM)	[224]
	Antioxidant activity by inhibiting superoxide anion radical in the hypoxanthine-xanthine oxidase system (IC_50_ = 3.0 μM)	[222]
Vescalin	Antibacterial activity against *Staphylococcus aureus* (MIC = 300 µg/mL)	[247]
Vescalagin	Antibacterial activity against *Staphylococcus aureus* (MIC = 400 µg/mL)	[247]
Castalin	Antibacterial activity against *Staphylococcus aureus* (MIC = 533 µg/mL)	[247]
Castalagin	Strong antibacterial activity against 20 strains of *Staphylococcus aureus* (MIC = 114 µg/mL), 26 strains of the genus *Salmonella* (MIC = 322 µg/mL) and *Vibrio vulnificus* (MIC = 83 µg/mL)	[240]
Komaniin	Chemopreventive activity as anti-proliferative activity in androgen-independent prostate cancer PC-3 cells (IC_50_ = 179.2 µM) and in androgen-dependent LNCaP cells (IC_50_ = 0.91 µM)	[224]
Punicafolin	Chemopreventive activity as anti-proliferative activity in androgen-independent prostate cancer PC-3 cells (IC_50_ = 314 µM) and in androgen-dependent LNCaP cells (IC_50_ = 0.68 µM)	[224]
Granatin B	Chemopreventive activity as anti-proliferative activity in androgen-independent prostate cancer PC-3 cells (IC_50_ = 157 µM) and in androgen-dependent LNCaP cells (IC_50_ = 2.06 µM)	[224]
Mallotusinic acid	Chemopreventive activity as anti-proliferative activity in androgen-independent prostate cancer PC-3 cells (IC_50_ = 308 µM) and in androgen-dependent LNCaP cells (IC_50_ = 3.48 µM)	[224]
**Proanthocyanidins**		
Procyanidin B1	Strong antioxidant activity by β-carotene destruction (0.126 mol/min)	[248]
	DNA damage repairability at a concentration of 50 μM promotes the development of mouse embryos in vitro by increasing OGG1 mRNA and protein expression in blastocysts	[249]
Procyanidin B3	Strong antioxidant activity by β-carotene destruction (0.126 mol/min)	[248]
Prodelphinidin B3 and C2	Anticancer activity through cell cycle arrest and caspase-3 activation in PC-3 prostate cancer cells (IC_50_ ˂ 50 µM)	[250]

MIC, minimal inhibitory concentration; IC_50_, half-maximal inhibitory concentration; TEAC, Trolox equivalent antioxidant capacity; ABTS, 2,2′-azino-bis(3-ethylbenzothiazoline-6-sulfonic acid); FRAP, ferric reducing antioxidant power; DPPH, 2,2-diphenyl-1-picrylhydrazyl; ORAC, oxygen radical absorbance capacity; PYR, pyranine; MRSA, methicillin-resistant *S. aureus*; HCMV, human cytomegalovirus; HCV, hepatitis C virus; DENV, dengue virus; MV, measles virus; RSV, respiratory syncytial virus; OGG1, 8-oxoguanine-DNA glycosylase-1.

## Data Availability

Not applicable.
